# Small Direct Inguinal Hernia, Lipoma of Spermatic Cord, Hydrocele, Parietal Hematocele, Cyst of Epididimys

**Published:** 1912-10

**Authors:** 


Small Direct Inguinal Hernia, Lipoma of Spermatic Cord, Hydrocele, Parietal Hematocele, Cyst of Epididimys. Vitrac and Chanaud, *Jour. de Med. de Bordeaux,* Feb. 4, 1912. The patient was a male aged 55, signs dating from 1885. Operation, cure. The main interest attaches to the number of complications.

